# Indole-3-Carbinol Induces Apoptosis in Human Osteosarcoma MG-63 and U2OS Cells

**DOI:** 10.1155/2018/7970618

**Published:** 2018-11-29

**Authors:** Chang Min Lee, Jongsung Lee, Myeong Jin Nam, See-Hyoung Park

**Affiliations:** ^1^Department of Life Science, Gachon University, Seongnam 13120, Republic of Korea; ^2^Department of Genetic Engineering, Sungkyunkwan University, Suwon 16419, Republic of Korea; ^3^Department of Bio and Chemical Engineering, Hongik University, Sejong 30016, Republic of Korea

## Abstract

This study was focused on investigating the anticancer potential of indole-3-carbinol (I3C) against osteosarcoma MG-63 and U2OS cells. A wound healing assay indicated that IC3 inhibited migration of MG-63 and U2OS cells. MTT, WST-1, and colony formation assays revealed that treatment of MG-63 and U2OS cells with I3C decreased cell viability. Fluorescence-activated cell sorting (FACS) analysis showed that I3C induced apoptosis in a dose- and time-dependent manner in MG-63 and U2OS cells. Moreover, via terminal deoxynucleotidyl transferase- (TdT-) mediated dUTP-biotin nick-end labeling (TUNEL) assay, we detected that I3C induced DNA fragmentation. Western blotting demonstrated that activated forms of caspase-3, caspase-7, and caspase-9, as well as poly (ADP-ribose) polymerase (PARP) were increased in MG-63 and U2OS cells, following treatment with I3C. Furthermore, protein expression levels of FOXO3, Bax, and Bim extra-large form were increased while those of Akt, JNK, p38, phosphorylated ERK, and Bcl-xL were decreased by I3C treatment in MG-63 and U2OS cells. Thus, the study indicates that I3C may induce apoptosis in human osteosarcoma MG-63 and U2OS cells via the activation of apoptotic signaling pathways by FOXO3.

## 1. Introduction

Osteosarcoma, the primary bone malignancy, is one of the most common cancers worldwide [1]. Generally, chemotherapy with agents such as cisplatin, methotrexate, and cyclophosphamide is widely used for treating osteosarcoma [2]. However, chemotherapy may result in drug resistance, as well as several side effects including drug-cytotoxicity which causes damage to normal tissues [3]. Therefore, alternative treatments for osteosarcoma need to be considered. At present, cancer-fighting foods are being discussed as potential therapeutic products against osteosarcoma. Daily intake of sufficient cancer-fighting foods is highly recommended by scientists. A typical example of a cancer-fighting food is tomato, considered a potential effector in prostate cancer treatment and prevention, because tomato contains lycopene which is a known anticancer compound [4]. Berries such as blueberries, raspberries, cherries, and strawberries are also recognized as antioxidant, antiaging, and anticarcinogenic foods [5]. Reportedly, berry fruits contain phenolic substances such as flavonoids and anthocyanins, which are recognized as anticancer agents [6].

Various nutritional and functional phytochemicals have been extracted from plants. Phytochemicals act as antioxidants by neutralizing free radicals which damage DNA, proteins and lipids [7]. These plant-derived substances also act as natural anticancer agents [8]. Phytochemicals have been used to treat many kinds of cancers such as breast, lung, colon, and liver cancer. Indole-3-carbinol (I3C) is a typical phytochemical contained in cruciferous vegetables such as cabbage, sprouts and broccoli [9]. I3C exerts anticancer effects on many kinds of cancers such as liver, lung, breast, colon, and prostate cancer [10–13]. However, the anticancer effects of I3C on human osteosarcoma have not been studied well. This study was focused on investigating the anticancer effects of I3C on human osteosarcoma MG-63 and U2OS cells. In this study, we especially focus on the activation of proapoptotic proteins such as caspase-3, caspase-7, and caspase-9, Bcl family and FOXO3.

Caspases are a protease enzyme family. Regulation of apoptosis is the main function of caspases [14]. Sequential activation of caspase family plays an important role in the execution of programmed cell death. Caspase-3, caspase-7, and caspase-9 are typical of caspase proteins that induce apoptosis in cells [15–17]. The current study evaluated the activation of caspase-3, caspase-7, and caspase-9 in I3C-treated MG-63 and U2OS cells. B-cell lymphoma-extra-large (Bcl-xL) is a transmembrane molecule found in mitochondria and is encoded by the Bcl-like 1 gene [18]. This protein induces activation of caspase, leading to apoptosis [19]. Bcl-2-like protein 11 (Bim), which is a member of the Bcl-2 protein family, is a proapoptotic protein [20]. Bax is also an essential executor of apoptosis [21]. In this study, we investigated the expression of Bcl family members such as Bcl-xL, Bim, and Bax.

Forkhead box (FOXO) families are transcription factors which are categorized by a specific fork head DNA-binding domain. FOXO transcription factors are involved in many signaling pathways and play crucial roles in many physiological processes [22]. Forkhead box O3 (FOXO3), which belongs to the forkhead family, is translocated from the nucleus into the cytoplasm after phosphorylation by the PI3K/Akt signaling pathway [23]. FOXO activates mitochondria-dependent and -independent apoptosis pathways [22]. Because regulation of FOXO3 is related to prevention of tumorigenesis, it is considered to be clinically significant. For example, translocation of* FOXO3* with the* MLL* is associated with the development of leukemia [24]. In this study, we investigated the involvement of FOXO3 in I3C-mediated apoptosis of MG-63 and U2OS osteosarcoma cells.

## 2. Materials and Methods

### 2.1. Reagents

I3C, purchased from Sigma-Aldrich (St. Louis, MO, USA), was dissolved in Dimethyl sulfoxide (DMSO, Sigma-Aldrich, St. Louis, MO) and 400 mM stock solutions of this preparation were stored at -20°C. EZ-Cytox was purchased from DoGenBio (Seoul, Korea). Caspase-3, caspase-7, caspase-9, and cleaved caspase-3, caspase-7, caspase-9, PARP, cleaved PARP, Akt, pAkt, Bcl-xL, Bim, Bad, Fas, and *β*-actin primary antibodies were obtained from Cell Signaling Technology (Danvers, MA). FOXO3, caspase-8, ERK, pERK, JNK, pJNK, p38, and pp38 primary antibodies were obtained from Santa Cruz Biotechnology (Santa Cruz, CA, USA). Anti-rabbit and anti-mouse secondary antibodies were purchased from Cell Signaling Technology.

### 2.2. Cell Culture

Human osteosarcoma MG-63 and U2OS cell lines were purchased from the American Type Culture Collection (ATCC, Manassas, VA, USA). These cells were incubated under standard conditions of 37°C, 5% CO_2,_ and 95% humidity. MG-63 and U2OS cells were cultured in Dulbecco's modified Eagle's medium (DMEM; GIBCO, Grand Island, NY, USA) containing 10% heat-inactivated (56°C, 30 min) fetal bovine serum and 1% antibiotics (penicillin/streptomycin).

### 2.3. Cell Proliferation Analysis by MTT Assay

MG-63 and U2OS cells were seeded in 96-well plates at a density of 3 × 10^3^ cells per well and allowed to attach for 24 h, respectively. Next, the cells were treated with an increasing concentration gradient of 0, 200, 400, and 600 *μ*M I3C, dissolved in DMSO (final concentration = 0.15%) and incubated for 24 and 48 h. Following the 24 and 48 h treatment, 20 *μ*L of MTT dye (5 mg/ml) were added to each well, after which the cells were incubated for 2 h at 37°C. After the supernatants were removed, formazan was solubilized in 200 *μ*L DMSO and absorbance was measured at 570 nm.

### 2.4. Cell Proliferation Analysis by WST-1 Assay

MG-63 and U2OS cells were seeded in 96-well plates at a density of 3 × 10^3^ cells per well and allowed to attach for 24 h, respectively. Then, the cells were treated with an increasing concentration gradient of 0, 200, 400 and 600 *μ*M 13C, dissolved in DMSO (final concentration = 0.15%) and incubated for 24 and 48 h. Following 24 and 48 h treatment, 10 *μ*L of EZ-Cytox were added to each well, after which the cells were incubated for 30 min at 37°C. After incubation, absorbance was measured at 450 nm.

### 2.5. Cell Proliferation Analysis by Colony Formation Assay

MG-63 (100/well) and U2OS (250/well) cells were seeded in 6-well plates and incubated for 24 h under standard conditions, respectively. After 24 h, the cells were treated with concentrations of 0, 200, and 400 *μ*M I3C for 24 h. The media was replaced with fresh media after 24 h and incubated for 2 weeks. The cells were washed with PBS twice for 3 min and fixed with 4% formaldehyde for 20 min at 4°C. Following fixation, the cells were again washed twice with PBS for 3 min and stained with 1% crystal violet (Sigma-Aldrich) solution for 30 min. The number of colonies was counted.

### 2.6. Wound Healing Assay

MG-63 and U2OS cells were seeded on six-well plates respectively. The next day, the cells were treated with different concentrations of I3C (DMSO, 200, 400, and 600 *μ*M) and a wound was artificially created by scraping with a pipette tip. The cells were rinsed twice with PBS and treated with media containing different concentrations of I3C. The wound area was observed for 1 day at 12 h intervals. Microscopic images of cells migrating to the wound area were obtained at 40x magnification using an inverted microscope (Olympus CKX53, Tokyo, Japan).

### 2.7. Terminal Deoxynucleotidyl Transferase- (TdT-) Mediated dUTP Nick-End Labeling Assay

Fluorescence of apoptotic cells was detected via terminal deoxynucleotidyl transferase- (TdT-) mediated dUTP nick-end labeling assay using the fluorometric TUNEL system (Promega, Madison, WI, USA). MG-63 and U2OS cells were plated on 6-well plates at a density of 3 × 10^5^ per well and incubated for 24 h, respectively. Cells were fixed with 4% formaldehyde for 25 min and permeabilized using 0.5% Triton X-100 for 10 min. Cells were then treated with 50 *μ*L TdT enzyme buffer. Cell nuclei were stained using 5 *μ*L Hoechst Stain Solution (Sigma-Aldrich). Labeled strand breaks were visualized using a fluorescence microscope (Nikon Eclipse TE 2000-U, Tokyo, Japan)

### 2.8. Annexin V/PI Staining

A FITC Annexin V apoptosis detection kit (BD Biosciences, Franklin Lakes, NJ, USA) was used to detect I3C-induced apoptosis. I3C-treated MG-63 and U2OS cells (3 × 10^5^ cells) were rinsed with PBS and suspended in 1× binding buffer. Cells were labeled with FITC Annexin V and incubated for 15 min at room temperature in the dark. Each cell was analyzed through flow cytometry (Beckman Coulter Brea, CA, USA)

### 2.9. Western Blot Analysis

MG-63 and U2OS cells (6 × 10^6^ cells, respectively) were treated with indicated doses of I3C, harvested and lysed using RIPA buffer (Sigma-Aldrich) containing protease and phosphatase inhibitors (Sigma-Aldrich). Cell lysate protein concentrations were measured using a Qubit™ Fluorocytometer (Invitrogen, Carlsbad, CA, USA). Proteins were separated via SDS-PAGE at 100 V for 2 h and transferred to an Immobilon-P transfer membrane (Merck Millipore, Burlington, MA, USA) to a nitrocellulose membrane at 45 V for 2 h. The membranes were blocked with bovine serum albumin (Bovogen, Australia), and incubated with primary antibodies against proteins at 4°C overnight. The membranes were then washed with TBS-T, and incubated using HRP-conjugated secondary antibody (Jackson Laboratory, Bar Harbor, USA). Chemiluminescence was detected using ECL (Gendepot, Barker, USA) and measured using the Chemi-doc detection system (Bio-Rad, Hercules, CA, USA)

### 2.10. Gelatin Zymography

Gelatin zymography was performed to detect the activities of matrix metalloproteinases (MMPs), especially MMP-2 and MMP-9. The cells were seeded in 100 mm cell culture plates (1.5 × 10^6^ cells/ml) and incubated under standard conditions for 24 h. Following incubation, the cells were treated with different concentrations of I3C (0, 200, 400* μ*M), containing 0.1% FBS, and incubated for 24 h under standard conditions. After incubation, the cultured media was harvested and concentrated using an Amicon Ultra-15 centrifugal filter (Millipore, Billerica, MA, USA). Media samples were mixed with SDS zymography sample buffer and separated via gelatin containing acrylamide gel. Gel was incubated in developing buffer (0.5 M Tris-HCL, 2 M sodium chloride, 50 mM calcium chloride) for 24 h. After developing, the gel was stained by Coomassie blue staining buffer.

### 2.11. Boyden Chamber Assay

Boyden chamber invasion assay was performed to evaluate the invasive ability of MG-63 and U2OS cells. The cells were seeded to the upper part of the Boyden chamber at a density of 5 × 10^4^ cells in 50 *μ*L of 0.1% FBS DMEM and 1% (MG-63) or 2.5% (U2OS) FBS DMEM containing different concentrations of I3C (0, 200, and 400 *μ*M) was loaded at the bottom part of the Boyden chamber. Gelatin-coated polycarbonate membrane with 8-*μ*m pore size was situated between the bottom and upper parts of the Boyden chamber. Following 3 h of incubation, MG-63 and U2OS cells that invaded the lower part of the membrane were stained with 1% crystal violet staining solution (Sigma-Aldrich). The numbers of invaded MG-63 and U2OS cells were counted.

### 2.12. Statistical Analysis

Statistical analysis was conducted using one-way ANOVA, and statistical significance was set at p < 0.05.

## 3. Results

### 3.1. I3C Reduced Migration Ability of MG-63 and U2OS Cells

**Figure 1:**
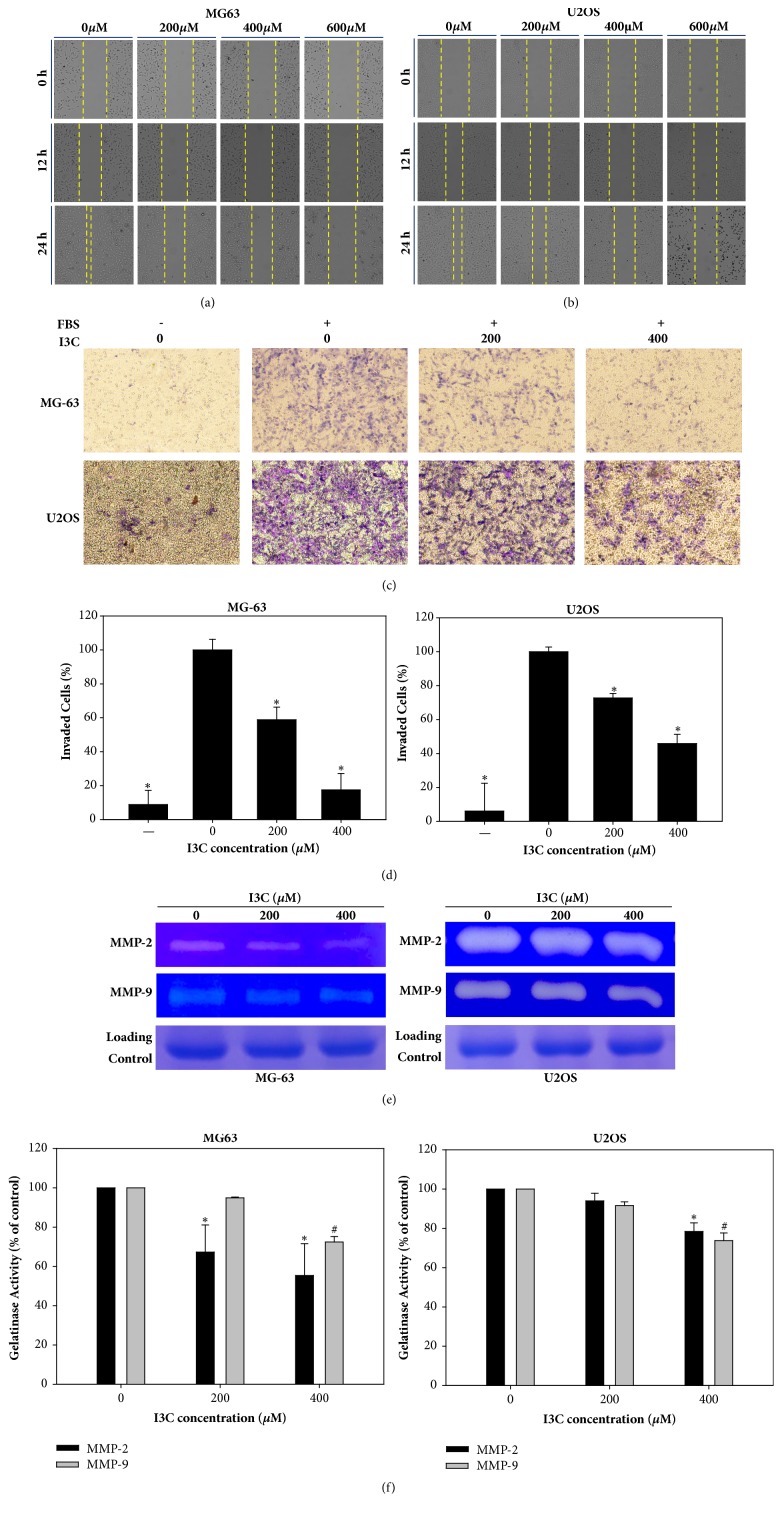
Inhibitory effect of I3C on migration of MG-63 and U2OS cells. Wound healing assay of I3C-treated MG-63 (a) and U2OS (b) cells. The number of cells migrating to the wound increased in vehicle-treated MG-63 and U2OS cells, whereas fewer cells migrated to the wound area in I3C-treated cells. This migration was dose-and time-dependent. (c) Invasion of MG-63 and U2OS cells was evaluated by Boyden chamber assay after treatment with different concentrations of I3C. (d) Invading cells were counted and the results were analyzed statistically using Student's t-test. *∗* = p<0.05. (e) Proteolytic activities of MMP-2 and MMP-9 were attenuated by the treatment of I3C in MG-63 and U2OS cells. (f) Activities of MMP-2 and MMP-9 were analyzed statistically using Student's t-test. *∗*, # = p<0.05.

Wound healing assay was conducted to evaluate wound healing ability in MG-63 and U2OS cells by the I3C treatment (Figures 1(a) and 1(b)). MG-63 and U2OS cell migration was significantly reduced following I3C-treatment. We observed that the wound area was healed in control cells. However, the proportion of cell migration decreased considerably in I3C-treated MG-63 and U2Os cells and filling of the wound area was dose-dependent. These results indicate that I3C treatment reduced the migration ability of MG-63 and U2OS cells. To investigate the effects of I3C on the migration ability of MG-63 and U2OS cells, Boyden chamber assay was performed. We found that the number of invading cells decreased following respective treatment of MG-63 and U2OS cells with 13C (Figure 1(c)). Statistical analysis indicated that the number of invading cells was significantly decreased in tomentosin-treated MG-63 cells compared to the control group (Figure 1(d)). Furthermore, gelatin zymography was performed to evaluate gelatinase activity of MMP-2 and MMP-9. Enzymatic activity of MMP-2 and MMP-9 decreased following treatment of MG-63 and U2OS cells with I3C (Figure 1(e)). Statistical analysis indicated that enzymatic activity of MMP-2 and MMP-9 were significantly inhibited after treatment with I3C. In summation, our results showed that migration ability of MG-63 and U2OS decreased following treatment with I3C (Figure 1(f)).

### 3.2. I3C Has Antiproliferative Effects in MG-63 and U2OS Cells

**Figure 2:**
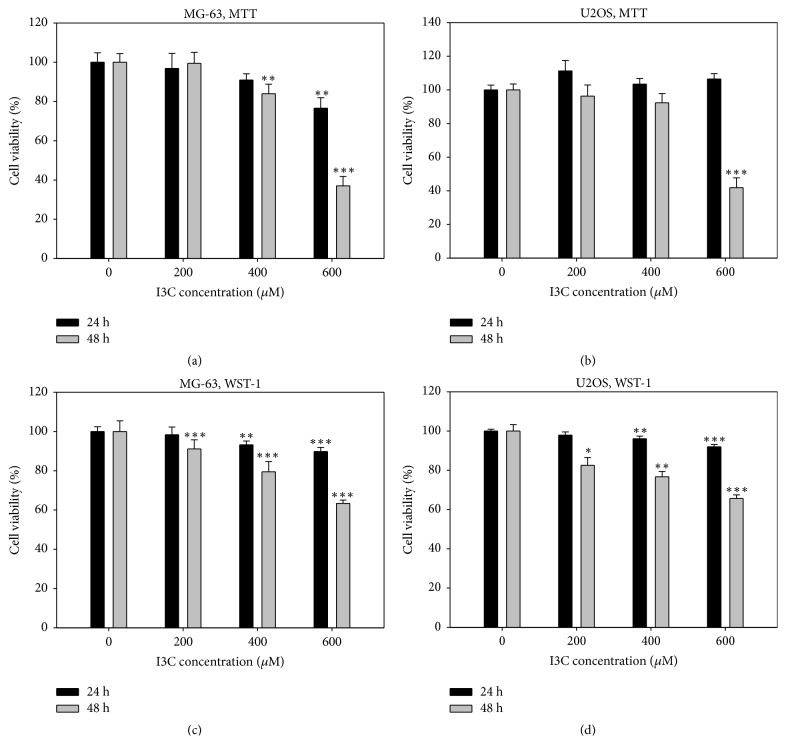
Cell cytotoxicity assay of MG-63 and U2OS cells treated with different concentrations of I3C ranging from 200-600 *μ*M at 24 h and 48 h time periods. Cell viability was evaluated by MTT and WST-1 assays. (a) Cell viability of MG-63 determined by MTT assay. (b) Cell viability of U2OS determined by MTT assay. (c) Cell viability MG-63 determined by WST-1 assay. (d) Cell viability of U2OS determined by WST-1 assay. The results were analyzed statistically using Student's t-test. *∗* = p < 0.05, *∗∗* = p < 0.01, and *∗∗∗* = p < 0.001.

**Figure 3:**
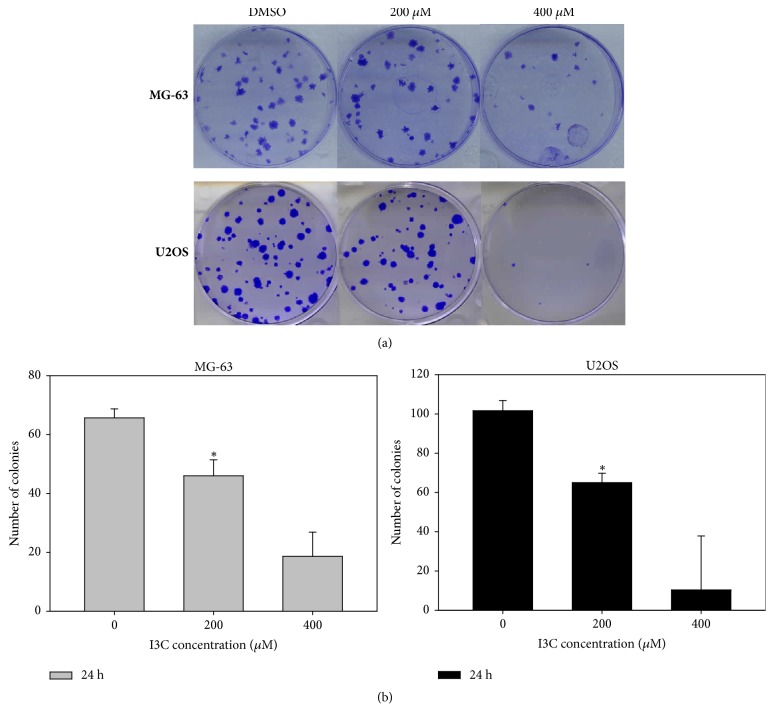
I3C suppresses the colony-forming ability of MG-63 and U2OS cells. The number of colonies was determined by the colony formation assay. (a) MG-63 (100 cells/well) and U2OS (250 cells/well) were treated with I3C (200 and 400 *μ*M) or the control (DMSO) for 1 day and media was replaced with fresh media (DMEM, 10% FBS, 1% antibiotics). After 2 weeks, the cells were stained with crystal violet solution. Representative images of colony formation assays are shown. (b) The number of colonies in I3C-treated wells was compared with that of the control group. The number of colonies was counted and the results were analyzed statistically using Student's t-test. *∗* = p<0.05.

To demonstrate antiosteosarcoma effects of I3C, we performed cell proliferation assays using the human osteosarcoma cell line MG-63 and U2OS. MG-63 and U2OS cells were treated with different concentrations of I3C (0, 200, 400, and 600 *μ*M), respectively for 24 and 48 h, followed by MTT and WST-1 assay analyses. MTT assay results showed that the antiproliferation effect of I3C on MG-63 and U2OS cells occurred in a dose- and time-dependent manner (Figures 2(a) and 2(b)). WST-1 assay results also indicated the antiproliferation effect of I3C on MG-63 and U2OS cells (Figures 2(c) and 2(d)). Colony formation assay was performed to evaluate antiproliferation effects of I3C on MG-63 and U2OS cells. The number of colonies decreased in a dose-dependent manner (Figures 3(a) and 3(b)). Our results showed that I3C may exert antiproliferation effects on human osteosarcoma MG-63 and U2OS cells.

### 3.3. I3C Induced Apoptosis in MG-63 and U2OS Cells

**Figure 4:**
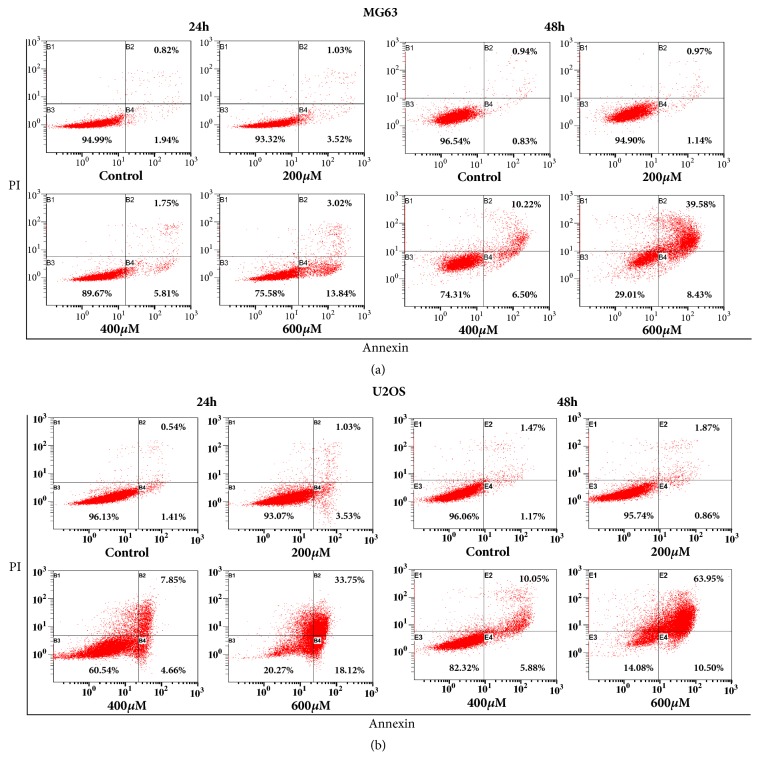
Evaluation of apoptosis in MG-63 and U2OS cells by Annexin V/PI double staining assay (flow cytometry) after 24 and 48 h of treatment with different concentration of I3C. I3C induced apoptosis in a dose-and time-dependent manner in MG-63 and U2OS cells. The percentage of apoptotic cells (B2 and B4) is shown. (a) MG-63 flow cytometry dot plots. (b) U2OS flow cytometry dot plots.

**Figure 5:**
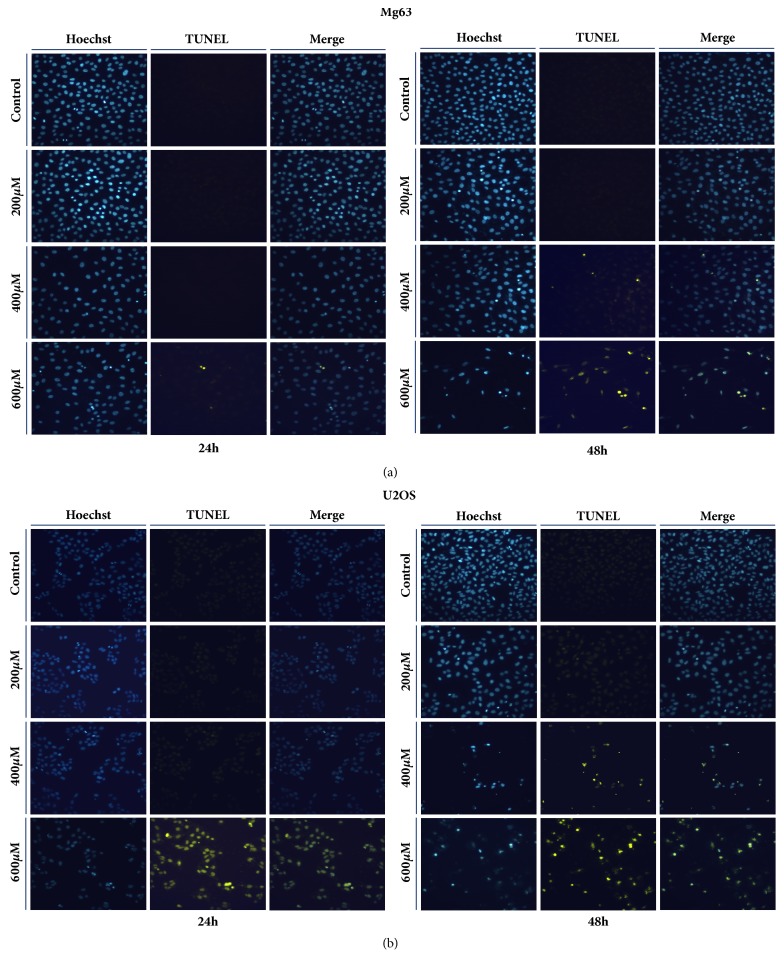
Detection of apoptosis by TUNEL assay in MG-63 (a) and U2OS (b) cells after 24 h and 48 h of treatment with different concentration of I3C. DNA fragmentations were detected using TUNEL assay in MG-63 and U2OS cells. Blue fluorescence shows nuclei, whereas green fluorescence shows DNA fragmentations. Blue-stained nuclei images and the green-stained fragmented DNA images were merged. The results showed that I3C induced DNA fragmentations in a dose- and time-dependent manner in MG-63 and U2OS cells.

**Figure 6:**
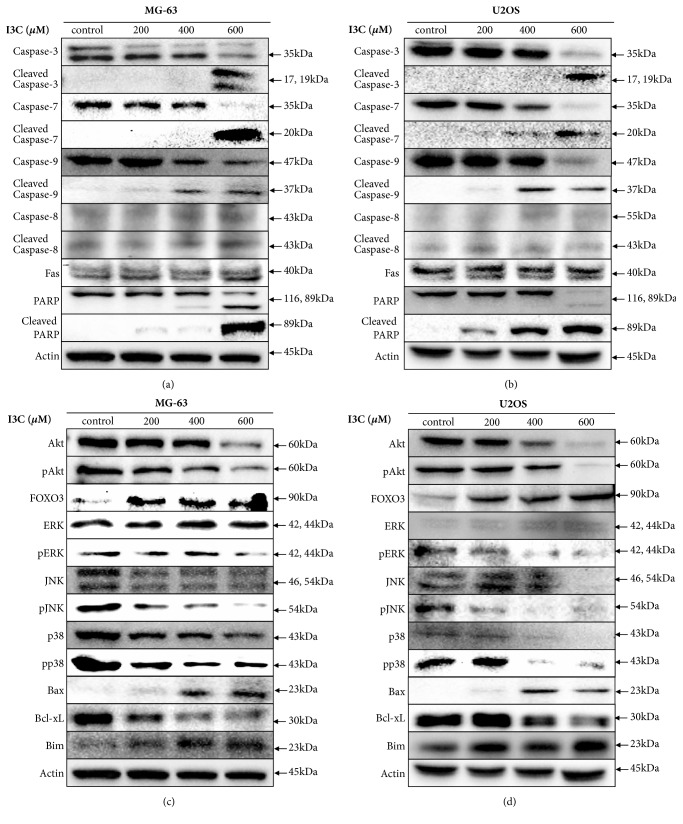
Western blot analysis of the dose-dependent effects of I3C in MG-63 and U2OS cells exposed to different concentration of I3C for 48 h. Caspase-3, caspase-7, caspase-8, caspase-9, Fas, and PARP expression in MG-63 (a) and U2OS (b) cells. FOXO3, Akt, JNK, ERK, p38, and apoptosis-related proteins expression in MG-63 (c) and U2OS (d) cells. *β*-actin was used as a gel-loading control.

We investigated whether the anticancer effect of I3C was associated with induction of apoptosis. Annexin V/PI staining analysis results indicated that increasing doses of I3C (0, 200, 400, and 600 *μ*M) for 24 and 48 h, induced apoptosis in MG-63 and U2OS cells in a dose- and time-dependent manner (Figures 4(a) and 4(b)). Apoptotic cells comprised 1.77% of the control group and 2.11%, 16.72%, and 48.01% of the 200, 400, and 600 *μ*M I3C treatment groups for 48 h on MG-63 cells, respectively. Apoptotic cells comprised 2.64% of the control group and 2.73%, 15.93%, and 74.45% of the 200, 400, and 600 *μ*M I3C treatment groups for 48 h on U2OS cells, respectively. Furthermore, we performed TUNEL assay to confirm whether I3C induced apoptosis in MG-63 and U2OS cells (Figures 5(a) and 5(b)). MG-63 and U2OS cells were incubated with 0, 200, 400, and 600 *μ*M of I3C for 24 h and 48 h. Next, control groups and I3C-treated groups were analyzed using the TUNEL assay. MG-63 and U2OS cells displaying green fluorescence increased with 13C treatment, suggesting that I3C-treatment induced DNA fragmentations in MG-63 and U2OS cells in a dose- and time-dependent manner (Figures 5(a) and 5(b)). Expression of apoptotic proteins was analyzed via western blotting to investigate the molecular mechanism of apoptosis in I3C-treated MG-63 and U2OS cells. Activation (increase of cleaved form) of caspase-3, caspase-7, caspase-9, and PARP in MG-63 and U2OS cells was induced by I3C (Figures 6(a) and 6(b)). We also evaluate the caspase-8, cleaved caspase-8, and Fas expression levels. We found that caspase-8 and cleaved caspase-8 expression levels were not changed significantly in MG-63 and U2OS cells. Moreover, Fas expression level was not changed significantly in MG-63 cells and U2OS cells. Taken together, these results suggest that I3C may induce apoptosis in osteosarcoma cells through caspase-3-, caspase-7-, and caspase-9-mediated mechanisms.

### 3.4. I3C Increased FOXO3 Expression for Apoptosis in MG-63 and U2OS Cells

In order to demonstrate the detailed working mechanism of apoptosis induced in U2OS cells by I3C treatment we performed western blotting analyses of a few oncogenic kinases and FOXO3 in MG-63 and U2OS cells treated with I3C. Expression of Akt, JNK, p38 and phosphorylated ERK was reduced by I3C treatment in a dose-dependent manner (Figures 6(c) and 6(d)). The expression of FOXO3 was markedly increased by I3C treatment in a dose-dependent manner. The expression of Bax and BimEL was also increased, while that of Bcl-xL was decreased by I3C treatment in a dose-dependent manner. These results suggested that I3C may downregulate Akt and phosphorylate ERK expression leading to the activation of FOXO3 transcriptional factor protein, which regulates Bax and BimEL in osteosarcoma cells.

## 4. Discussion

Despite recent developments in diagnostic technology and therapy, medical doctors and researchers have been unable to find an effective method to treat osteosarcoma, due to drug resistance and metastasis. Researchers have explored new agents or molecules that may be used to treat various cancers including osteosarcoma effectively. Osteosarcoma is the most common type of pediatric bone tumor [25]. Reportedly, incidence of pediatric osteosarcoma may be higher than 10% [26]. Osteosarcoma is divided into three forms based on grade as low, middle and high grade osteosarcoma [27]. Low grade osteosarcoma patients have a good prognosis with a 91% five-year survival rate following surgery alone [28]. However, in the case of middle grade osteosarcoma, chemotherapy is not very effective and the benefits of additional chemotherapy have not been established [29, 30]. High grade osteosarcoma accounts for approximately 80% of osteosarcomas. Chemotherapy before or after surgery may improve patient survival rates to 60%, which is higher than that by surgery alone [31]. Although surgery combined with chemotherapy has been developed, over 30% of osteosarcoma patients develop metastatic cancer in other tissues, leading to a poor prognosis for these patients. Survival time of metastatic cancer patients is less than one year [32]. Thus, more effective therapeutic options are required in the case of osteosarcoma.

It is well known that specific caspases (caspase-8, caspase-9, and caspase-10 in humans) function as upstream “initiators” of apoptosis, by activating downstream “effector” caspases (caspase-3 and caspase-7) [33]. Caspase-3 plays a crucial role in morphological changes and DNA fragmentations associated with apoptosis [34]. Caspase-7 and caspase-9 also play an important role in the execution of programmed cell death. Caspase-3 is an activator of apoptotic DNA Fragmentation [35]. Caspase-7 activation also induces morphological and biochemical changes including exposure of phosphatidylserine and genomic DNA fragmentation [16]. Activation of caspase-3 and caspase-7 induced cell death in monocytic THP-1 cells and activation of PARP induced apoptosis in C6 glioma cells [36, 37]. Activation of caspase-9 is mediated by the Apaf-1 apoptosome and caspase-9 induces apoptosis through caspase-3 activation in human cervical cancer cells [38, 39]. We detected that treatment of I3C increased the cleaved of caspase-3, caspase-7, and caspase-9 in MG-63 and U2OS cells. These results indicated that I3C may induce caspase-3-, caspase-7-, and caspase-9-mediated apoptotic effects in MG-63 and U2OS cells. Poly (ADP-ribose) polymerase (PARP) is a protein related to cellular processes such as DNA repair and apoptosis [40]. PARP is an important factor in genomic stability, and caspase-mediated cleavage of PARP is a biochemical hallmark of apoptosis [41, 42]. In the current study, it was observed that treatment with 13C induced cleavage of PARP in MG-63 and U2OS cells. These results demonstrated that I3C may exert a PARP-mediated apoptotic effect on MG-63 and U2OS cells.

Reportedly, FOXO transcription factors play critical roles in many physiological and pathological conditions such as cancer [43]. FOXO3 is a member of FOXO family and a multispecific transcription factor which is responsible for many transcriptional processes such as cell proliferation and programmed cell death [44]. Translocalization of FOXO3 to the nucleus induces FOXO3-mediated apoptosis [45]. Since FOXO3A is known to play a pivotal role in inducing tumor suppression through apoptosis and cell cycle arrest of a number of cancer cells, we investigated whether the cells treated with I3C would undergo apoptosis via upregulation of FOXO3A. Recently, He et al., demonstrated that Casticin, a flavone, may show anticancer activity by inhibiting hepatocellular carcinoma cell growth through G(2)/M cell cycle arrest [46]. They demonstrated that the FOXO3A-p27Kip1 pathway in hepatocellular carcinoma cell lines was activated by treatment with Casticin, as well as downregulation of FOXM1 which decreases FOXO3A expression. Our findings indicated that the level of FOXO3 expression was increased in MG-63 and U2OS cells. Activation of various apoptotic proteins such as BimEL and Bax was also observed. Downregulation of Bcl-xL induces apoptosis through Bax activation in human pancreatic cancer cells [47]. Bax translocation to mitochondria is also an important factor in apoptosis [21]. Our results also indicated that I3C induced the downregulation of Bcl-xL in MG-63 and U2OS cells.

Mitogen-activated protein kinases (MAPKs) control various physiological processes. Extracellular signal-regulated kinases (ERKs) play an essential role in cell division [48]. ERKs activate either extrinsic or intrinsic apoptosis pathways by releasing cytochrome c from mitochondria to the cytoplasm or by activating caspase-8 [49]. However, no significant ERK expression changes occurred in I3C-treated MG-63 and U2OS cells. C-Jun amino-terminal kinases (JNKs) and p38 MAPKs are also essential mediators of cell proliferation, differentiation, migration, and survival [50]. JNKs play a crucial role in regulating mitochondrial apoptotic proteins [51]. JNK activates apoptotic signaling pathways by upregulating proapoptotic genes [51]. However, JNK also displays antiapoptotic functions depending on cell type, and activation of other signaling [52]. We investigated JNK and p38 MAPK expressions in I3C-treated MG-63 and U2OS cells. Findings showed that JNK and p38 MAPK expression decreased in I3C-treated MG-63 and U2OS cells. Moreover, regulation of the Akt/Bad pathway also induces mitochondria-mediated apoptosis in human hepatocellular carcinoma HepG2 cells [53].

Akt protein regulates the function of various substrates involved in cell proliferation, cell cycle progression and cell growth [54]. In recent years, it has been reported that PI3K/Akt signaling pathway components are changed in various human cancers [54]. Inactivation of Akt signaling caused cancer cell death. AT7867 inhibited AKT-S6K1 signaling pathways in human colon cancer HT-29 cells [55]. Moreover, inhibition of Akt activity was identified as the main molecular events responsible for CTet activity in human breast cancer cells MCF-7 and p53-mutant MDA-MB-231 cells [56]. Interestingly, we observed the significant degradation of Akt protein After 600 *μ*M of I3C treatment for 48 h in MG-63 and U2OS cells. The results suggested that Akt is an important mediator of tumor proliferation in Mg-63 and U2OS cells.

I3C is a compound found in cruciferous vegetables, such as cabbage, broccoli, and cauliflower [57]. Recent studies showed that I3C may exert anticancer effects on various cancer cells, such as colon, liver, and lung cancer via the regulation of apoptotic signaling proteins [11, 13, 58]. Typical mechanism underlying apoptosis induced by I3C proceeds via the translocation of Bax protein to mitochondria in breast cancer cells [59]. I3C also inhibits cell proliferation and induces G1 cell cycle arrest in prostate cancer cells [60]. However, anticancer effects of I3C on osteosarcoma have not been studied well. The aim of this study was to investigate the anticancer effect of I3C on human osteosarcoma MG-63 and U2OS cells. To the best of our knowledge, this is the first report demonstrating that upregulation of FOXO3 signaling pathway by I3C may be involved in inducing apoptosis in osteosarcoma cells. Animal experiments using mouse xenograft model with human osteosarcoma cells are planned to support our current results and provide an experimental basis for a novel antiosteosarcoma therapeutic strategy. Taken together, these results suggest that the anticancer activity of I3C treatment may rest on the induction of FOXO3-mediated apoptotic signaling pathways, and its application may lead to a novel therapeutic agent for osteosarcoma. In conclusion, our research demonstrated that I3C upregulates the expression of proapoptotic cleaved PARP and cleaved caspase-3, caspase-7, and caspase-9 and downregulates the expression of antiapoptotic Bcl-xL.

## Data Availability

All of data used to support the findings of this study are included within the article.
